# Global phosphoproteomics reveals DYRK1A regulates CDK1 activity in glioblastoma cells

**DOI:** 10.1038/s41420-021-00456-6

**Published:** 2021-04-16

**Authors:** Ariadna Recasens, Sean J. Humphrey, Michael Ellis, Monira Hoque, Ramzi H. Abbassi, Brianna Chen, Mitchell Longworth, Elise J. Needham, David E. James, Terrance G. Johns, Bryan W. Day, Michael Kassiou, Pengyi Yang, Lenka Munoz

**Affiliations:** 1 Charles Perkins Centre and School of Medical Sciences, Faculty of Medicine and Health, The University of Sydney, Camperdown, NSW 2006 Australia; 2 Charles Perkins Centre and School of Life and Environmental Sciences, Faculty of Science, The University of Sydney, Camperdown, NSW 2006 Australia; 3 School of Chemistry, Faculty of Science, The University of Sydney, Camperdown, NSW 2006 Australia; 4 Oncogenic Signalling Laboratory, Telethon Kids Institute, Perth Children’s Hospital, 15 Hospital Avenue, Nedlands, WA 6009 Australia; 5 QIMR Berghofer Medical Research Institute, 300 Herston Road, Herston, QLD 4006 Australia; 6 Charles Perkins Centre and School of Mathematics and Statistics, Faculty of Science, The University of Sydney, Sydney, NSW 2006 Australia; 7 Computational Systems Biology Group, Children’s Medical Research Institute, University of Sydney, Westmead, NSW 2145 Australia

**Keywords:** Cell biology, Cancer

## Abstract

Both tumour suppressive and oncogenic functions have been reported for dual-specificity tyrosine phosphorylation-regulated kinase 1A (DYRK1A). Herein, we performed a detailed investigation to delineate the role of DYRK1A in glioblastoma. Our phosphoproteomic and mechanistic studies show that DYRK1A induces degradation of cyclin B by phosphorylating CDC23, which is necessary for the function of the anaphase-promoting complex, a ubiquitin ligase that degrades mitotic proteins. DYRK1A inhibition leads to the accumulation of cyclin B and activation of CDK1. Importantly, we established that the phenotypic response of glioblastoma cells to DYRK1A inhibition depends on both retinoblastoma (RB) expression and the degree of residual DYRK1A activity. Moderate DYRK1A inhibition leads to moderate cyclin B accumulation, CDK1 activation and increased proliferation in RB-deficient cells. In RB-proficient cells, cyclin B/CDK1 activation in response to DYRK1A inhibition is neutralized by the RB pathway, resulting in an unchanged proliferation rate. In contrast, complete DYRK1A inhibition with high doses of inhibitors results in massive cyclin B accumulation, saturation of CDK1 activity and cell cycle arrest, regardless of RB status. These findings provide new insights into the complexity of context-dependent DYRK1A signalling in cancer cells.

## Introduction

Dual-specificity tyrosine phosphorylation-regulated kinase 1A (DYRK1A) is activated by autophosphorylation of tyrosine residues during protein translation. Thus, the mature DYRK1A protein is a constitutively active kinase whose signalling via phosphorylation of downstream substrates at serine/threonine residues is regulated largely by DYRK1A expression levels, known as the dosage effect^1^. DYRK1A has been implicated in a diverse variety of biological processes, including CNS development, Down syndrome, beta-cell proliferation and diabetes, as well as Alzheimer’s disease^1–6^. However, the role of DYRK1A in cancer is not fully understood, and both oncogenic and tumour suppressive roles for DYRK1A have been reported^7,8^. For example, DYRK1A functions as a tumour suppressor in breast cancer and acute myeloid leukaemia^9,10^. In contrast, some evidence suggests oncogenic roles for DYRK1A, via stabilization of receptor tyrosine kinases^7,11^ and acceleration of tumour growth^12,13^. Further adding to the DYRK1A controversy, DYRK1A inhibitors have been repeatedly reported to exert anti-proliferative activity when tested on cancer cells^14–17^. Off-target effects are well-known players in the anti-cancer efficacy of kinase inhibitors^18^ and kinome screens demonstrate that DYRK1A inhibitors potently inhibit other kinases^3–6^. Nevertheless, a recent study using six orthogonal DYRK1A inhibitors demonstrated that the off-target effects are redundant with respect to the proliferative effects of DYRK1A inhibitors, at least in pancreatic beta cells^4^.

Glioblastoma is a lethal brain tumour characterized by molecular heterogeneity and aberrant cellular hierarchy. At the apex of this hierarchy sits a small population of glioblastoma stem cells, which are key mediators of tumour propagation and therapy failure^19^. Inhibition of DYRK1A in glioblastoma stem cell induces degradation of epidermal growth factor receptor (EGFR), resulting in decreased self-renewal of EGFR-addicted cells^7^. In contrast, evidence suggests that elevated expression of DYRK1A leads to destabilization of hypoxia-inducible factor 2α, loss of glioblastoma stemness and inhibition of tumour growth^8^. Furthermore, DYRK1A-regulated DREAM assembly and quiescence were established in glioblastoma cells where DYRK1A was ectopically over-expressed^20^. Collectively, while these observations make it clear that DYRK1A is an important kinase in glioblastoma, they also suggest that the function of DYRK1A in glioblastoma, and probably in other tumours, is dependent on the cell type and its genetic background.

In this study, we delineate a novel DYRK1A signalling pathway in glioblastoma stem cells. Phosphoproteomic and mechanistic studies revealed that DYRK1A regulates the anaphase-promoting complex (APC) ligase complex and degradation of cyclin B, the primary co-activator of the CDK1. We confirmed that DYRK1A inhibition leads to CDK1 activation. Importantly, we discovered that the proliferative response to CDK1 activation upon DYRK1A inhibition is determined by the retinoblastoma (RB) expression and the degree of residual DYRK1A activity.

## Results

### Phosphoproteomics reveals that DYRK1A regulates CDK1

**Fig. 1:**
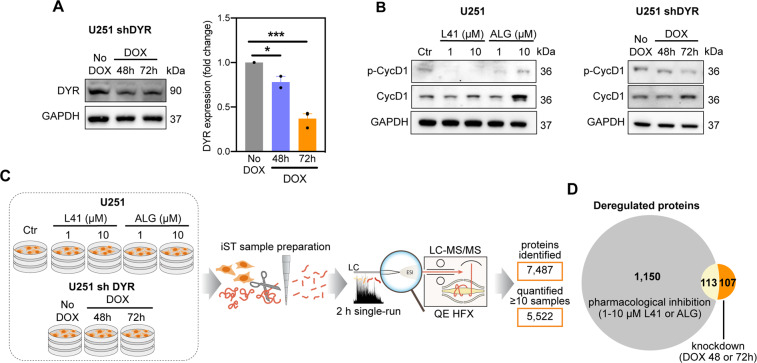
Proteomic characterization of DYRK1A inhibition in U251 glioblastoma cells. **A** DYRK1A (DYR) expression in U251 cells transfected with doxycycline (DOX)-inducible shRNA targeting DYRK1A (U251 shDYR). Representative images and densitometric quantification of three independent experiments are shown (mean ± SEM, unpaired *t*-test, *P < 0.05; ***P < 0.001). **B** Expression of phosphorylated (p-CycD1) and total cyclin D1 (CycD1) in U251 cells treated ± DYRK1A inhibitors L41 and ALGERNON (ALG) for 72 h, and in U251 shDYR cells treated ± DOX. Representative images of two independent experiments are shown. **C** Design and summary of the proteomic analysis of U251 cells treated ± L41 and ALG for 72 h, and U251 shDYR cells treated ± DOX. **D** Venn diagram showing overlap among proteins deregulated after DYRK1A pharmacological inhibition (1263 proteins total) and genetic knockdown (220 proteins total).

To obtain a global view of DYRK1A-regulated pathways, we analysed the proteomes and phosphoproteomes of U251 glioblastoma cells infected with doxycycline-inducible DYRK1A shRNA (Fig. 1A) or treated with DYRK1A inhibitors leucettine 41 (L41, IC_50_ = 40 nM)^21^ and ALGERNON (ALG, IC_50_ = 77 nM)^22^. Target engagement was confirmed by decreased phosphorylation of cyclin D1 (Fig. 1B), a direct DYRK1A downstream substrate^23^. Single-run proteome measurements identified ~7400 proteins (Fig. 1C and Table S1) with high reproducibility (Pearson correlation > 0.98 for all samples; Fig. S1A). Globally, DYRK1A inhibitors changed expression levels of 1200 proteins, while knockdown of DYRK1A changed levels of 220 proteins (Figs. 1D and S1B). Around 50% of the proteins affected by DYRK1A knockdown were also affected by inhibitors, while only 10% of those affected by inhibitors were also affected by knockdown (Fig. 1D). This suggests that DYRK1A inhibitors likely exhibit some off-target effects.

**Fig. 2:**
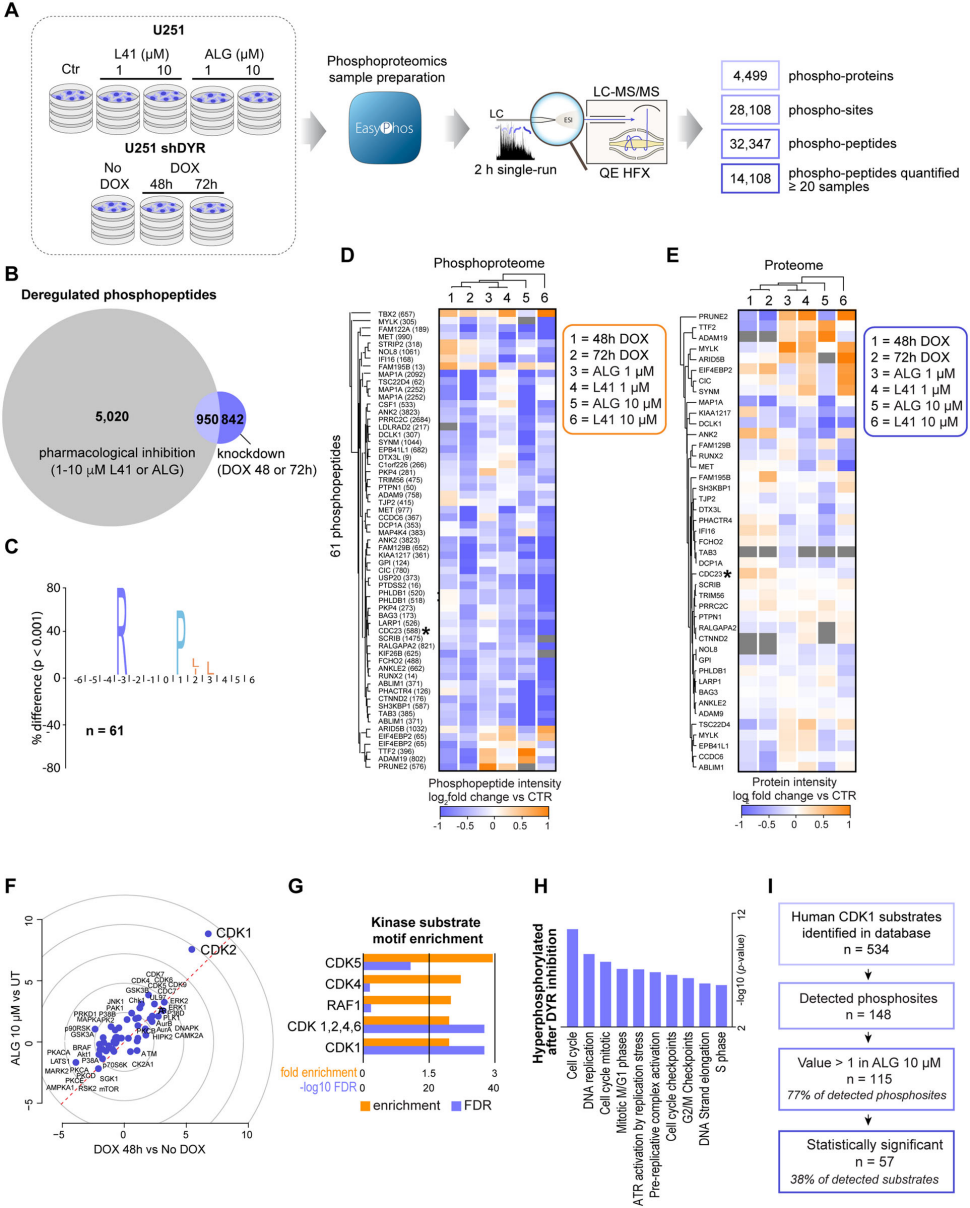
Phosphoproteomic characterization of DYRK1A inhibition in U251 glioblastoma cells reveals CDK1 hyperactivity. **A** Design and summary of the phosphoproteomic analysis of U251 cells treated ± DYRK1A inhibitors L41 and ALGERNON (ALG) for 72 h, and U251 shDYR cells treated ± DOX. **B** Venn diagram showing overlap among phosphopeptides significantly (ANOVA adj. *P* < 0.05 and Dunnett’s *P* < 0.05) reregulated by DYRK1A pharmacological inhibition (5,970 total) and genetic knockdown (1792 total). **C** A consensus sequence preference for DYRK1A. Motif enrichment analysis of 61 phosphopeptides regulated by both pharmacological and genetic inhibition that also possess an Arginine (R) in the –3 position, reveals a strong preference for Proline (P) in the +1 position. **D** Heatmap of phosphopeptide intensities (median log2 fold changes for treatment *vs* their respective controls) for phosphopeptides identified as potential DYRK1A substrates. **E** Heatmap of protein changes (median log2 fold changes of protein LFQ intensities for treatments *vs* their respective controls) for proteins on which phosphosites from panel D are found. **F** Kinase perturbation analysis with Kinase PA showing relative kinase activity in *z*-scores from DOX 48 h *vs* No DOX treatments (*x*-axis) and ALG (10 μM) vs untreated treatments (*y*-axis). **G** Kinase-substrate motif enrichment analysis for sites hyperphosphorylated in response to DYRK1A (DYR) inhibition. **H** Gene ontology over-representation analysis (Fischer’s exact test) of significantly hyperphosphorylated proteins in cells treated with ALG (10 μM, 72 h). **I** Annotation of CDK1 substrates (derived from PhosphoSitePlus) in the phosphoproteome of cells treated with ALG (10 μM, 72 h).

To quantify changes to signalling of glioblastoma cells induced by DYRK1A inhibition, we employed the EasyPhos phosphoproteomics workflow^24,25^. In untreated cells, we quantified over 28,000 phosphorylation sites located on 4499 proteins (Fig. 2A). Globally, 84% of phosphorylation sites were localized to a single amino acid with high precision (median localization probability 0.99; Table S2). Quantification coverage was high, with over 15,000 phosphorylation sites quantified in each sample with high reproducibility (Figs. 2A and S2A).

As with the proteome, DYRK1A inhibitors exerted more pronounced effects on the phosphoproteome than did DYRK1A knockdown (Fig. S2B), and we observed similar overlap in regulated phosphorylation events in response to each treatment type (Fig. 2B). While phosphorylation sites that are unique to pharmacological inhibition may include off-target effects of these inhibitors, sites overlapping with DYRK1A knockdown are likely enriched in bona fide DYRK1A substrates. To explore phosphorylation sites regulated by both pharmacological and genetic DYRK1A inhibition, we performed an analysis of the amino acid sequences surrounding phosphorylated residue, revealing a strong preference for Proline (P) in the +1 position (Fig. S2C). DYRK1A is a basophilic kinase^26^, and in agreement with this, we observed an enrichment of Arginine (R) in the −3 position of these phosphorylation sites. Taking this as a *‘minimal consensus sequence’* filter and applying it to the 950 commonly regulated sites highlighted 61 phosphorylation sites that may represent putative DYRK1A substrates (Fig. 2C, D). Of these 61 phosphorylation sites, almost all were decreased in several treatment conditions compared to control samples (Fig. 2D) and were not decreased at the protein level (Fig. 2E), strengthening our hypothesis that these may be enriched in direct DYRK1A substrates.

Analysis of sites hyperphosphorylated upon 10 µM ALG and DYRK1A knockdown revealed an enrichment of CDK and RAF1 kinase-substrate motifs (Fig. 2F, G). On a pathway level, we observed enrichment of cell cycle, DNA replication and mitotic processes (Fig. 2H). Given the robust substrate motif enrichment for CDK1/2 (Fig. 2F), we investigated phosphorylation changes in CDK1 substrates in more detail. From a total of 534 reported CDK1 substrates (www.phosphosite.org), 148 were present in our phosphoproteomic dataset. Of these, 115 (77%) displayed increased phosphorylation after DYRK1A inhibition, with 37 of these being significantly hyperphosphorylated (Fig. 2I and Table S3). These analyses suggest for the first time that DYRK1A inhibition leads to CDK1 activation.

### DYRK1A regulates cyclin B degradation

**Fig. 3:**
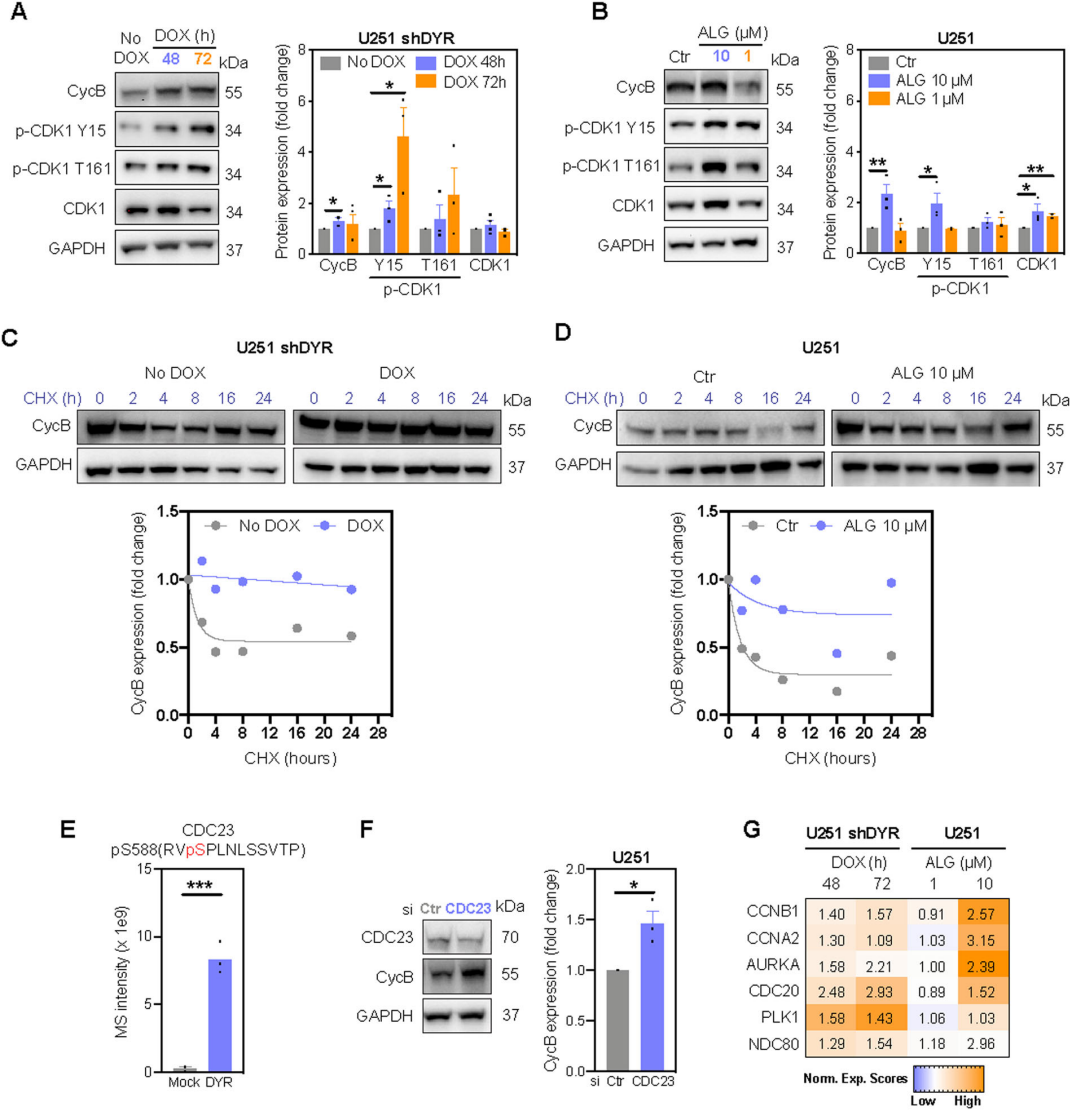
DYRK1A inhibition prevents cyclin B degradation. Immunoblot analysis and densitometric quantification of cyclin B (CycB), phosphorylated (p-CDK1) and total CDK1 in (**A**) U251 shDYR cells treated ± DOX and (**B**) U251 cells treated ± DYRK1A inhibitor ALGERNON (ALG, 72 h). Degradation of cyclin B (CycB) in U251 shDYR cells treated ± DOX (**C**) and U251 cells treated ± ALG (**D**). Cells were treated with DOX or ALG for 72 h before cycloheximide (CHX) treatment. Representative images and quantification from two independent experiments are shown. **E** Mass spectrometry (MS) intensity of CDC23 phosphorylated at S588 by DYRK1A in vitro. **F** Immunoblot analysis and densitometric quantification of cyclin B expression in cells transfected with siRNA targeting CDC23 (72 h). **G** Heatmap of protein levels of anaphase-promoting complex substrates cyclin B1 (CCNB1), cyclin A2 (CCNA2), Aurora kinase A (AURKA), cell-division cycle protein 20 (CDC20), Polo-like kinase 1 (PLK1) and kinetochore protein NDC80 in U251 cells following doxycycline-induced DYRK1A knockdown (DOX) and inhibition with ALG (72 h). Data represent fold change of replicates to their respective controls. All bar graphs represent mean ± SEM of at least three independent experiments (two-tailed unpaired *t*-test; *P < 0.05, **P < 0.01, ***P < 0.001).

CDK1 activity is controlled by precise regulation of cyclin B levels and CDK1 phosphorylation. Phosphorylation of CDK1 on residue T161 induces the conformational changes necessary for CDK1 activity, while T14 and Y15 phosphorylation inactivate CDK1^27^. Our MS data revealed ~2-fold increased cyclin B levels, as well as hyperphosphorylation of CDK1 on Y15 and T161 in response to DYRK1A inhibition (Tables S1 and S2), which was confirmed by immunoblotting (Fig. 3A, B). Phosphorylation of the inhibitory Y15, despite the fact that CDK1 activity increased upon DYRK1A inhibition (Fig. 2F, G), is consistent with previous work showing that substantial CDK1 activity can occur concomitantly with high levels of CDK1 Y15 phosphorylation^27,28^. We also observed elevated, but not statistically significant CDK1 phosphorylation at the activating T161 upon DYRK1A inhibition. Nevertheless, our MS data did not reveal any changes in the expression or activity of kinases known to phosphorylate CDK1 at Y15 (Wee1, Myt1) and at T161 (CAK1)^29^ (Tables S1 and S2). Thus, given that CDK1 phosphorylation did not justify increased CDK1 activity, we concluded that DYRK1A inhibition likely leads to CDK1 activation by increasing cyclin B levels. In support, DYRK1A inhibition prevented degradation of cyclin B in U251 cells (Fig. 3C, D).

Cyclin B is primarily degraded by the APC composed of 11–13 proteins^30^. Phosphoproteomic analysis identified the APC subunit CDC23 as one of novel DYRK1A substrates, with CDC23 phosphorylation at S588 decreasing upon DYRK1A inhibition (Fig. 2D, asterix). In vitro kinase assays using recombinant proteins confirmed phosphorylation of CDC23 at S588 by DYRK1A (Figs. 3E and S3). Given that CDC23 phosphorylation is crucial for APC formation^31^, we propose that decreased CDC23 phosphorylation after DYRK1A inhibition impairs proper functioning of the APC. Indeed, CDC23 knockdown increased the levels of cyclin B (Fig. 3F), and levels of additional APC substrates were increased in the proteome of U251 cells upon DYRK1A inhibition (Fig. 3G).

### DYRK1A regulates cell proliferation in a dose-dependent manner

**Fig. 4:**
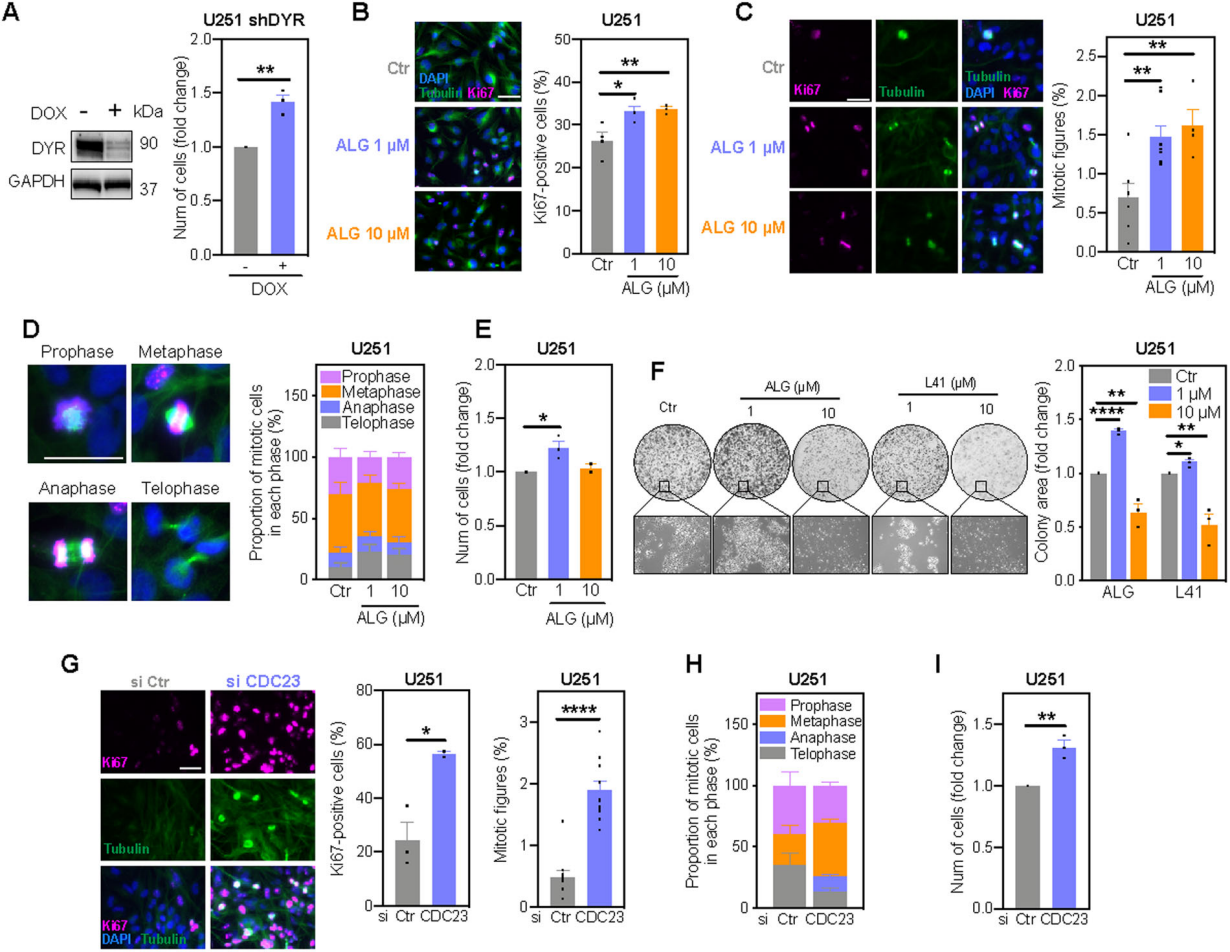
DYRK1A inhibition alters proliferation of U251 glioblastoma cell in a dose-dependent manner. **A** DYRK1A (DYR) expression (72 h) and nuclear staining quantification (7 days) of U251 shDYR cells treated ± DOX. Immunofluorescence images and quantification of Ki67-positive cells (**B**) and mitotic figures (**C**) in U251 cells treated with Algernon (ALG) for 7 days. Cells were stained using antibodies against Ki67 (pink) and tubulin (green). DAPI was used to visualize cell nuclei (blue). **D** Immunofluorescence images of mitotic phases and quantification of mitotic cells in Figure 2C for representation of each mitotic phase individually. **E** Nuclear staining quantification of cells treated with ALG for 7 days. **F** Clonogenic survival (round images), appearance (rectangle images) and quantification of colonies in cells treated with ALG and L41 for 14 days. **G** Immunofluorescence images and quantification of Ki67-positive cells and mitotic figures in U251 cells transfected with siRNA targeting CDC23 (7 days). Cells were stained using antibodies against Ki67 (pink) and tubulin (green). DAPI was used to visualize cell nuclei (blue). **H** Mitotic cells in Figure 2G were analysed for representation of each mitotic phase individually. **I** Nuclear staining quantification of cells transfected with siRNA targeting CDC23 (7 days). All bar graphs represent mean ± SEM of at least three independent experiments (two-tailed *t*-test; *P < 0.05, **P < 0.01, ***P < 0.001). Scale bars: 5 μm.

Since the CDK1/cyclin B complex is the master regulator of mitosis and pathway analysis indicates activation of mitosis upon DYRK1A inhibition (Fig. 2H), we next investigated the effect of DYRK1A inhibition on U251 cell proliferation. DYRK1A knockdown significantly increased cell numbers over 7 days (Fig. 4A). Similarly, the DYRK1A inhibitor ALG (1 μM) increased the number of Ki67-positive cells and mitotic figures (Fig. 4B, C), especially during late mitotic telophase (Fig. 4D), resulting in increased cell numbers and colony formation (Fig. 4E, F). Intriguingly, although 10 μM ALG increased cyclin B levels to a greater extent than the 1 μM treatment (Fig. 3B), we did not observe a further increase in mitosis or proliferation (Fig. 4B–F). Microscopic images revealed the presence of morphologically normal cells, suggesting a cytostatic effect of ALG at 10 μM (Fig. 4F). Similar findings were obtained using the orthogonal DYRK1A inhibitor L41 (Fig. 4F). Given that saturation of CDK1 activity achieved with non-degradable cyclin B leads to mitotic arrest^32,33^, these data suggest that complete DYRK1A inhibition results in cell cycle arrest due to immense accumulation of cyclin B and potentially, other mitotic regulators (Fig. 3G).

In support of the DYRK1A-CDC23 axis controlling glioblastoma cell cycle, CDC23 knockdown increased the percentage of Ki67-positive cells, the number of mitotic figures and overall cell numbers (Fig. 4G–I). Thus, CDC23 knockdown phenocopied the effects of DYRK1A knockdown and low-dose ALG (Fig. 4A–F). Taken together, the proliferative response to DYRK1A inhibition, cyclin B accumulation and consequent CDK1 activation is dose-dependent (Fig. 4). While partial DYRK1A inhibition by knockdown (protein levels reduced by ~60%) and low-dose inhibitors increased proliferation, complete DYRK1A inhibition with high-dose inhibitors results in cell cycle arrest.

### DYRK1A inhibition regulates proliferation in a cell-dependent manner

**Fig. 5:**
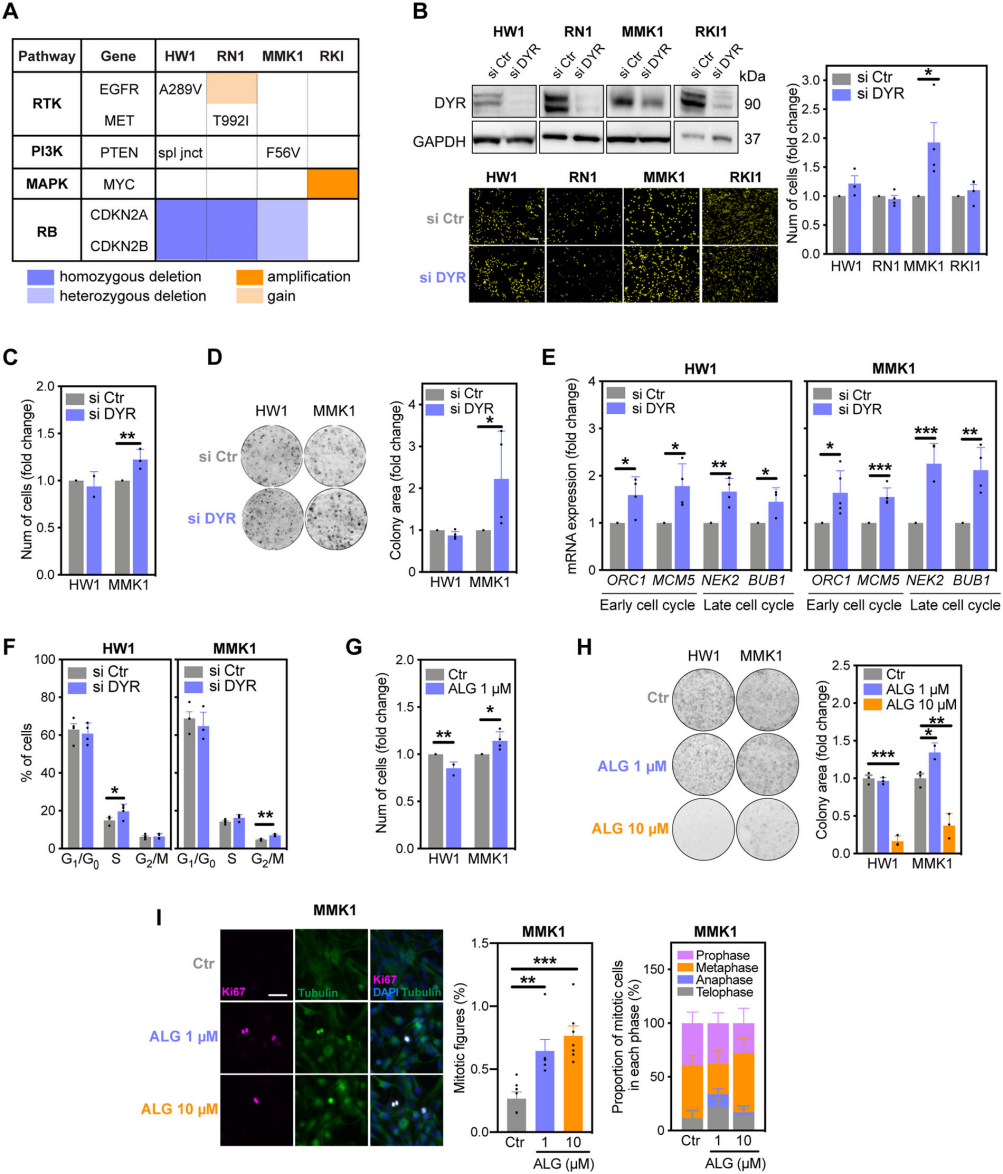
DYRK1A inhibition regulates proliferation in a cell-dependent manner. **A** Genomic profiles of glioblastoma stem cell lines HW1, RN1, MMK1 and RKI. **B** Immunoblot analysis (72 h) and nuclear staining quantification (7 days) of cells transfected with scramble (si Ctr) and DYRK1A-targeting (si DYR) siRNAs. Scale bar: 10 μm. **C** Manual counting of cells transfected with si Ctr and si DYR for 7 days. **D** Representative images and quantification of colonies in cells transfected with si Ctr and si DYR for 14 days. **E** RT-PCR analysis of cell cycle genes in cells transfected with si Ctr and si DYR for 7 days. **F** Flow cytometry quantification of cells in G1/G0, S and G_2_/M phases following transfection with si Ctr and si DYR for 3 days. **G** Nuclear staining quantification of cells treated with DYRK1A inhibitor ALGERNON (ALG) for 7 days. **H** Representative images and quantification of colonies in cells treated with ALG for 14 days. **I** Immunofluorescence images and quantification of mitotic figures in cells treated with ALG for 7 days. Cells were stained using antibodies against Ki67 (pink) and tubulin (green). DAPI was used to visualize cell nuclei (blue). Scale bar: 5 μm. All bar graphs represent mean ± SEM from at least three independent experiments (two-tailed, unpaired *t*-test, *P < 0.05, **P < 0.01, ***P < 0.001).

To investigate DYRK1A-CDK1 pathway in clinically relevant models, we knocked down DYRK1A in four patient-derived glioblastoma stem cell lines (Fig. 5A)^34^. Surprisingly, DYRK1A knockdown only increased proliferation in MMK1 cells, leaving the proliferation rates of HW1, RN1 and RKI1 cells unchanged (Fig. 5B). Further assays confirmed that DYRK1A knockdown increased MMK1 proliferation, whereas the proliferation rate of HW1 cells remained unchanged (Fig. 5C, D). Silencing DYRK1A increased the expression of S-phase (*ORC1, MCM5*) and mitotic (*NEK2, BUB1*) genes in both HW1 and MMK1 cell lines (Fig. 5E). Mitotic genes were upregulated to a greater extent in MMK1 cells, where we also observed increased proliferation (Fig. 5B–D). Flow cytometry revealed that DYRK1A knockdown increased accumulation of HW1 cells in S-phase (from basal 15% to 21.4%), whereas MMK1 cells accumulated in G_2_/M-phase (from basal 4.8% to 7.1%; Fig. 5F). Consistent with DYRK1A knockdown data, low-dose ALG (1 μM) increased cell numbers, the number of mitotic figures and colony growth in MMK1 but not in HW1 cells (Fig. 5G–I). High-dose ALG (10 μM) failed to further increase proliferation in MMK1 cells and showed cytostatic effects (Fig. 5H), in agreement with the results obtained in U251 cells. Thus, partial DYRK1A inhibition increased proliferation only in MMK1 cells, implying that the molecular profiles of cells shape the proliferative response to DYRK1A inhibition.

### Retinoblastoma expression determines the proliferative response to DYRK1A inhibition

**Fig. 6:**
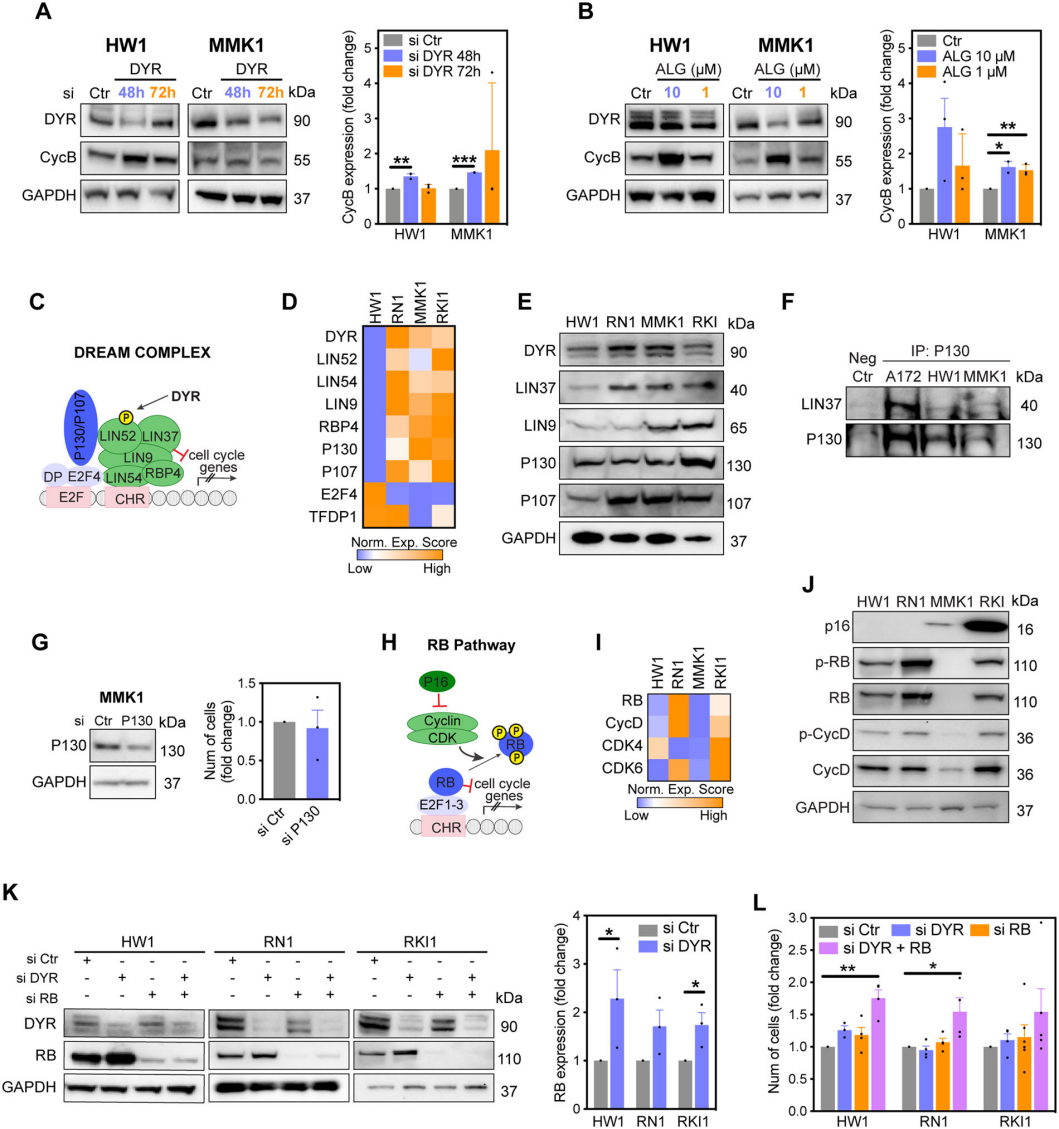
Retinoblastoma expression determines the proliferative response to DYRK1A inhibition. **A, B** Immunoblot analysis and densitometric quantification of cyclin B (CycB) expression in cells treated with scramble (si Ctr) and DYRK1A (si DYR) targeting siRNA (72 h), and in cells treated with DYRK1A inhibitor ALGERNON (ALG, 72 h). **C** Schematic overview of DREAM complex. RNA seq data showing gene expression (**D**) and immunoblot analysis showing protein expression (**E**) of DREAM complex components in glioblastoma stem cells. **F** Immunoblot analysis of DREAM complex assembly in glioblastoma cells. Cell lysates were first immunoprecipitated (IP) with an anti-P130 antibody, then immunoblotted against P130 and LIN37. **G** Immunoblot analysis of MMK1 cells transfected with P130-targeting siRNA (si P130) for 72 h and nuclear staining quantification of cells transfected with si P130 for 7 days. **H** Schematic overview of retinoblastoma (RB) pathway. RNA seq data (**I**) and immunoblot analysis (**J**) of RB pathway components in glioblastoma stem cells. **K**, **M** Immunoblots analysis of cells transfected with scramble (si Ctr), DYRK1A-targeting (si DYR) and RB-targeting (si RB) siRNA for 72 h. Densitometric quantification of RB expression following DYRK1A knockdown is shown. **L** Nuclear staining quantification of cells single transfected with si DYR or si RB, and double transfected (si DYR + RB) for 7 days. All bar graphs represent mean ± SEM from at least three independent experiments (two-tailed unpaired *t*-test, *P < 0.05, **P < 0.01, ***P < 0.001).

To further investigate different proliferation responses to DYRK1A inhibition, we first analysed the DYRK1A-cyclin B axis. DYRK1A silencing increased cyclin B levels in both MMK1 and HW1 cells (Fig. 6A, B), suggesting that additional mechanism controls the proliferative response to DYRK1A inhibition. We next investigated assembly of the DREAM complex, through which DYRK1A regulates G_0_ cell cycle arrest (Fig. 6C)^20^. RNA sequencing^34^ and immunoblotting revealed that expression of DREAM proteins (low in HW1, RN1; high in MMK1, RKI1 cells; Fig. 6D, E) did not correlate with increased proliferation rates in MMK1 cells. Furthermore, we found that the DREAM complex is not assembled in glioblastoma stem cells, and knockdown of the key DREAM component P130 had no effect on MMK1 cell proliferation (Fig. 6F, G). DREAM assembly in A172 glioblastoma cells (Fig. 6F) served as a positive control for immunoprecipitation experiments^35^.

Next, we investigated compatibility of the RB pathway. HW1 and RN1 cells carry homozygous *CDKN2A/B* deletion, whereas MMK1 and RKI1 cells carry heterozygous deletion and intact *CDKN2A/B*, respectively (Fig. 5A). *CDKN2A* encodes the CDK inhibitor p16 that inhibits CDK4/6-dependent RB phosphorylation (Fig. 6H). RNA-seq revealed higher expression of RB pathway components in RN1 and RKI1 cells compared to HW1 and MMK1 cells (Fig. 6I). Analysis of protein levels confirmed the absence of p16 in HW1 and RN1, low p16 expression in MMK1 and high levels in RKI1 cells (Fig. 6J). Protein levels of RB and cyclin D1 mirrored their mRNA profiles and confirmed that MMK1 cells lack RB protein (Fig. 6I, J).

Considering that RB-deficient MMK1 cells increased their proliferation rate upon DYRK1A inhibition (Fig. 5B), we hypothesized that RB compensates for DYRK1A loss in cell cycle regulation. Thus, we performed single and double RB and DYRK1A knockdown in RB-proficient cells (Fig. 6K). While single knockdown of either RB or DYRK1A did not change the proliferation rates, double knockdown significantly increased cell proliferation (Fig. 6L). Furthermore, DYRK1A knockdown increased RB expression by 2-fold (Fig. 6K). In summary, DYRK1A knockdown increased the expression of cyclin B in glioblastoma stem cells, regardless of their molecular background. However, this cyclin B accumulation selectively increased the proliferation rate of RB-deficient cells. In RB-proficient cells, cyclin B accumulation in response to DYRK1A inhibition appears to be neutralized by increased RB expression, resulting in unchanged proliferation rate.

### DYRK1A inhibition reactivates the cell cycle in dormant RB-deficient cells

**Fig. 7:**
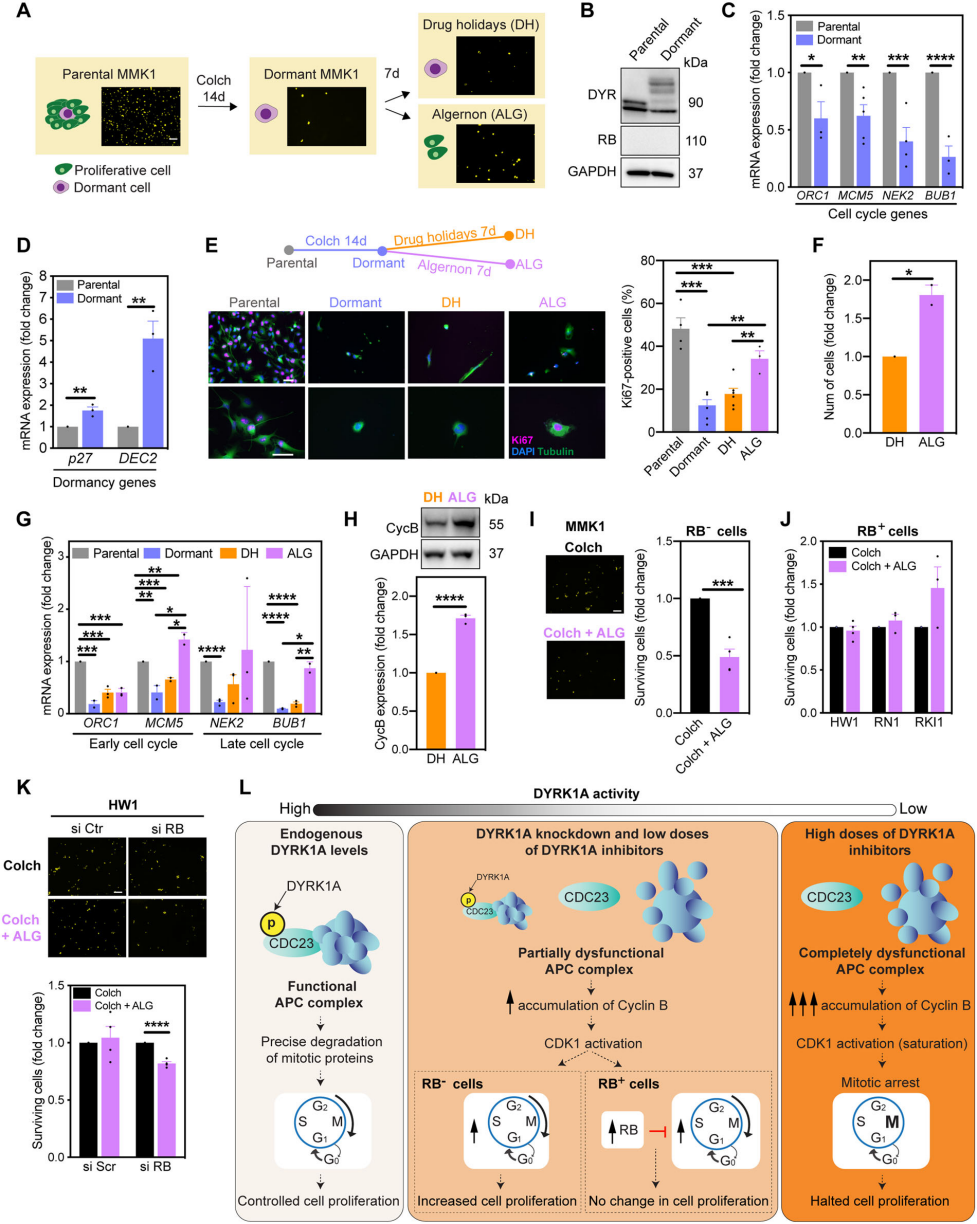
DYRK1A inhibition reactivates the cell cycle in dormant RB-deficient cells. **A** Schematic overview of the experimental design. Dormant MMK1 cells were isolated by treating parental MMK1 cell population with 0.5 μM of colchicine (Colch) for 14 days. Dormant cells were then cultured in drug-free medium (drug holidays, DH) or treated with DYRK1A inhibitor ALGERNON (ALG). Scale bar: 10 μm. **B** Immunoblot analysis of DYRK1A (DYR) and retinoblastoma (RB) expression in parental and dormant MMK1 cells. Representative images of two independent experiments are shown. **C**, **D** RT-PCR analysis of cell cycle and dormant genes in dormant MMK1 cells. **E** Immunofluorescence images and quantification of Ki67-positive MMK1 cells. Dormant cells were treated ± ALG (1 μM, 7 days), then stained to detect Ki67 (pink), tubulin (green) and DAPI (blue). Scale bar: 5 μm. **F** Nuclear staining quantification of dormant MMK1 cells treated ± ALG (1 μM) for 7 days. **G** RT-PCR analysis of cell cycle genes in MMK1 cells treated as outlined in panel (**E**). **H** Immunoblot analysis and densitometric quantification of cyclin B (Cyc B) expression in dormant MMK1 cells treated ± ALG (1 μM) for 72 h. **I**, **J** Images and nuclear staining quantification of cells treated with Colch ± ALG (1 μM) for 14 days. Scale bar: 10 μm. **K** Images and nuclear staining quantification of HW1 cells treated with Colch ± ALG (1 μM) for 14 days. Prior to drug treatments, cells were transfected with scramble (si Ctr) and RB (si RB) targeting siRNA (24 h). Scale bar: 10 μm. **L** Summary of the proposed role of DYRK1A in glioblastoma stem cell proliferation. Bar graphs represent mean ± SEM from at least three independent experiments (two-tailed unpaired *t*-test; *P < 0.05, **P < 0.01, ***P < 0.001, ****P < 0.0001).

Finally, we questioned whether DYRK1A inhibition reactivates the cell cycle in dormant cancer cells, which create a pool of drug-tolerant persister cells surviving anti-proliferative drugs^36^. To isolate dormant cells, RB-deficient MMK1 cells were treated for 14 days with high concentration (25x EC_50_ from 5-day viability assay) of the microtubule-targeting agent colchicine (Fig. 7A)^37^. Expression of DYRK1A and lack of RB in dormant cells remained unchanged, although DYRK1A appears to be post-translationally modified in dormant cells, which changes its molecular weight (Fig. 7B). Cell cycle genes were decreased and dormancy genes were increased in dormant cells (Fig. 7C, D). Furthermore, dormant cells resumed proliferation upon colchicine removal (i.e. drug holidays), demonstrating a reversible dormancy phenotype (Fig. S4). To study the effect of DYRK1A inhibition on cell dormancy, dormant MMK1 cells were treated with a proliferation-promoting low-dose ALG (1 μM) for 7 days (Fig. 7E). Consistent with our data in parental MMK1 cells (Figs. 5 and 6), ALG increased the percentage of Ki67-positive cells and overall cell numbers (Fig. 7E, F). Seven days of drug holidays initiated re-expression of cell cycle genes (Fig. 7G, orange bars) necessary for cell cycle recovery (Fig. S4). This effect was significantly enhanced by addition of ALG (Fig. 7G, pink bars), accompanied with increased cyclin B expression (Fig. 7H).

Many chemotherapeutics, including the experimental drug colchicine used herein, predominantly kill actively cycling cells, while sparing dormant cells^36^. Our findings that DYRK1A inhibition forced cells to leave the dormant state prompted us to investigate whether DYRK1A inhibitors could improve the efficacy of anti-proliferative drugs. Indeed, co-treatment with colchicine and ALG (1 μM) reduced the number of dormant MMK1 cells compared to colchicine treatment alone (Fig. 7I). Lastly, we addressed RB and DYRK1A cooperativity in dormant glioblastoma cells. Given that DYRK1A inhibition did not increase proliferation in RB-proficient HW1, RN1 or RKI1 cells (Fig. 5B), we expected and confirmed that ALG did not decrease the number of colchicine-surviving dormant cells (Fig. 7J). Importantly, RB knockdown in HW1 cells combined with colchicine + ALG significantly reduced the number of dormant cells (Fig. 7K). These observations further demonstrate the cooperativity between RB and DYRK1A in cell cycle regulation.

## Discussion

In this study, we demonstrate for the first time that DYRK1A negatively regulates mitosis. Thus, our work adds DYRK1A to the small category of kinases that function as tumour suppressors, such as PKC and MKK4^38,39^. Moreover, we identified 61 putative, novel DYRK1A downstream targets and validated CDC23 as a bona fide DYRK1A substrate. We report that partial DYRK1A inhibition increases proliferation and reactivates cell cycle of dormant RB-deficient glioblastoma cells, thereby improving efficacy of anti-proliferative drugs. Finally, we report that complete DYRK1A inhibition causes cell cycle arrest.

Mechanistically, we linked DYRK1A inhibition to the accumulation of cyclin B, which in turn activated CDK1, a key regulator of mitosis. We propose a model in which DYRK1A phosphorylates CDC23, an essential subunit for the proper function of the APC ligase^31^. CDC23, CDC27 and CDC16 form the scaffolding tetratricopeptide repeat subcomplex of the APC, which is necessary for substrate recognition^30^. Disrupting the formation of this subcomplex via targeting any of its subunits prevents recruitment and ubiquitination of cyclin B^40^. Similar to our results with DYRK1A and CDC23 knockdown leading to cyclin B accumulation, CDC27 inhibition also attenuated cyclin B degradation^41^. In addition to controlling APC ligase, DYRK1A regulates the E3 ligase RNF169^42,43^ and Von Hippel-Lindau ligase^8^ and we identified ligases DTX3l and TRIM56 as putative DYRK1A targets (Fig. 2D), suggesting that DYRK1A is an upstream regulator of the proteasome.

Inhibition of DYRK1A causes activation of mitotic CDK1 regardless of the transcriptomic profile of the cell. However, a particularly striking observation was that this CDK1 activation specifically affects proliferation of RB-deficient cells and does not work in RB-expressing cells. On a systems level, this indicates that RB and DYRK1A cooperatively regulate the cell cycle. RB functions as a break in early G_0_ and G_1_ phases^44^, whereas DYRK1A slows down mitosis via cyclin B degradation (shown in this study). Given that single knockdown of RB or DYRK1A did not impact the cell proliferation rates, whereas knockdown of both DYRK1A and RB enhanced proliferation, it appears that DYRK1A is redundant to the presence of RB and vice versa. Thus, it is plausible that RB status is responsible for the variable proliferation phenotypes of DYRK1A targeting reported within the same histological cancer types^7,12,35^.

In addition to RB status impacting the behaviour of DYRK1A-targeted cells, we also observed that the cellular response to DYRK1A inhibition is dose-dependent. While inhibition of DYRK1A systematically increased cyclin B levels in a dose-dependent fashion, only knockdown reducing DYRK1A levels by ~60% and low-dose DYRK1A inhibitors increased proliferation. In contrast, DYRK1A inhibitors at high concentrations, despite further increasing cyclin B and CDK1 activity, had a cytostatic effect. Consistent with our findings, dose–response curves for DYRK1A inhibitors revealed increasing rates of pancreatic beta-cell proliferation up to a given dose, after which proliferation declines^4^. Given that saturation of CDK1 activity with non-degradable cyclin B leads to mitotic arrest^32,33^, we propose that complete DYRK1A inhibition with high concentrations of DYRK1A inhibitors and consequent massive accumulation of cyclin B also results in CDK1 saturation and cell cycle arrest (Fig. 7L). This conclusion explains the anti-proliferative effects of DYRK1A inhibitors reported by numerous studies^7,12,14–17,35^. Inhibitors used in these studies inhibit DYRK1A with biochemical IC_50_ below 100 nM, and cellular target engagement has been demonstrated at 1 μM^14–17^. Yet, in cell-based experiments, DYRK1A inhibitors were used at 10–30 μM concentration^7,12,35^. Finally, we show that low-dose DYRK1A inhibitors reactivate the cell cycle of dormant cells, thereby improving the efficacy of anti-proliferative drugs. While DYRK1A inhibition enhanced the apoptotic efficacy of imatinib by disrupting DREAM complex in quiescent gastrointestinal cancer cells^45^, in glioblastoma cells, the anti-dormancy efficacy of DYRK1A inhibition is a result of CDK1 activation and is effective only in RB-deficient cells.

In summary, we delineated a novel DYRK1A-CDK1 signalling pathway. Importantly, we have also established that the phenotypic response to DYRK1A inhibition depends on both RB status and the degree of residual DYRK1A activity (Fig. 7L). Moderate DYRK1A inhibition is required to enhance proliferation in RB-deficient cells. In contrast, complete DYRK1A inhibition results in the saturation of CDK1 activity and cell cycle arrest, regardless of RB status. These findings provide new insights into the complexity of DYRK1A signalling in cancer cells.

## Material and methods

### Cell culture

A172 and U251 cells were obtained from the European Collection of Cell Cultures (EACC, UK) in 2014 and authenticated in 2020 by Cell Bank Australia using short tandem profiling. Cells were cultured in DMEM supplemented with 10% FBS and Antibiotic-Antimycotic solution (Life Technologies, Carlsbad, CA, USA) at 37 °C and 5% CO_2_. Patient-derived HW1, RN1, MMK1 and RKI1 glioblastoma stem cells^34^ were cultured in KnockOut DMEM/F-12 supplemented with StemPro NSC SFM, 2 mM GlutaMAX-ICTS, 20 ng/mL EGF, 10 ng/mL FGF-β and Antibiotic-Antimycotic solution (all Life Technologies) as adherent cells on flasks coated with MatriGel (Corning, NY, USA). The protocols were approved by the Human Ethics Committee of the Royal Brisbane & Women’s Hospital (RBWH 2004/161). Cell cultures were routinely tested for mycoplasma infection and culturing did not exceed 15 passages.

### Doxycycline-inducible DYRK1A knockdown

Recombinant DNA Plasmid: sense 5′-cccGTTCGGCTTGCACCGTCATTTCtcgaGAAATGA CGGTGCAAGCCGAACttttttc-3′; antisense 5′-tcgagaaaaaaGTTCGGCTTGCACCGTCATTTC tcgaGAAATGACGGTGCAAGCCGAAC-3′. The DYRK1A inducible knockdown was developed using a lentiviral single vector system containing an H1-tetO-shRNA cassette and the codon-optimized tetR linked to EGFP^46^. shRNA for DYRK1A was developed into plasmids via *E. coli* transformation. Vector packaging system containing the shRNA-DYRK1A (10 µg/µL) was transfected via calcium phosphate precipitation method^47^ into HEK293T cells in the presence of 25 µM chloroquine. Concentrated lentivirus was added to U251 cells in a complete ‘Tet-free’ media containing 4 μg/mL Polybrene^®^ for 16 h. Infected live cells were sorted for GFP-positivity by flow cytometry using a BD FACS Influx (purity > 95%). Cells were maintained in 100 μg/mL normocin for 7 days and hairpin expression was induced via treatment with 10 µg/mL doxycycline.

### Synthesis and characterization of ALGERNON (ALG)

ALG was synthesized as previously reported^22^ and tested in-house using a radioactive DYRK1A kinase assay^17^. Residual DYRK1A activity in the presence of newly synthesized ALGERNON (1 μM) was 36.3 ± 4% (*n* = 3).

### Phosphoproteome sample preparation

Cells were lysed in SDC buffer, heated (5 min, 95 °C), cooled on ice and sonicated. A 10 µL aliquot was diluted 1:5 in 8 M urea and protein concentration was determined with BCA assay. Then, 200 µg of protein was diluted in SDC buffer, alkylated with 10 mM Tris(2-carboxyethyl)phosphine/40 mM 2-chloroacetamide pH 8 (5 min, 45 °C), digested with 1:100 Lys-C and Trypsin overnight at 37 °C with agitation (1500 rpm). Phosphopeptides were enriched using the EasyPhos workflow^24^. Eluted phosphopeptides were dried in a SpeedVac concentrator (Eppendorf) and resuspended in MS loading buffer (0.3% TFA/ 2% acetonitrile) prior to LC-MS/MS measurement.

### MS-based DYRK1A kinase assay

CDC23 (100 ng; Novus Biological, Centennial, CO, USA) was incubated with DYRK1A (30 ng, prepared in-house^17^) in 25 µL reaction buffer (50 mM HEPES pH 7.5, 1 mM EGTA, 0.01% Tween 20, 10 mM MgCl_2_, 1 mM dithiothreitol) and 100 µM ATP (90 min, 30 °C). Reactions were terminated by a 10 min incubation at 70 °C. The protein was reduced and alkylated with 10 mM TCEP / 40 mM CAA pH 8 (5 min, 45 °C). Urea (final 2 M) was added and proteins were digested with 5 ng of Lys-C and Trypsin (18 h, 30 °C). Peptides were desalted on StageTips containing styrene divinylbenzene-reverse phase sulfonate, eluted, dried and resuspended in MS loading buffer (0.3% TFA/2% acetonitrile) prior to LC-MS/MS measurement.

### LC-MS/MS measurements and data processing

Peptides and phosphopeptides were loaded onto a 40 cm column (75 µm inner diameter) fused silica packed with 1.9 µM C18 ReproSil particles (Dr. Maisch GmBH) and maintained at 60 °C. A Dionex U3000 RSLC Nano HPLC system (Thermo Fisher Scientific) was interfaced with a Q Exactive HF X benchtop Orbitrap mass spectrometer using a NanoSpray Flex ion source (Thermo Fisher Scientific). Peptides were separated with a binary buffer system of 0.1% (v/v) formic acid (buffer A) and 80% (v/v) acetonitrile / 0.1% (v/v) formic acid (buffer B). For phosphoproteome and in vitro kinase assay analysis, peptides were eluted at 350 nL/min and separated with a gradient of 3–19% buffer B over 40 min, followed by 19–41% buffer B over 20 min. Peptides were analysed with a full scan (350–1400 *m/z*; *R* = 60,000 at 200 *m/z*) at a target of 3e6 ions, followed by up to ten data-dependent MS2 scans using HCD (target 1e5 ions; max. IT 50 ms; isolation window 1.6 *m/z*; NCE 27%; min. AGC target 2e4), detected in the Orbitrap mass analyser (*R* = 15,000 at 200 *m/z*). Dynamic exclusion (30 s) and Apex trigger (2–4 s) were switched on. For single-run proteome analysis, peptides were eluted at 300 nL/min, separated with a gradient of 5–30% buffer B over 2 h and analysed with a full scan (350–1400 *m/z*; *R* = 60,000 at 200 *m/z*) at a target of 3e6 ions, followed by up to 20 data-dependent MS2 scans using HCD (target 1e5 ions; max. IT 28 ms; isolation window 1.4 *m/z*; NCE 27%; min. AGC target 1e4), detected in the Orbitrap mass analyser (*R* = 15,000 at 200 *m/z*). Dynamic exclusion (30 s) was switched on. Raw MS data were processed using MaxQuant^48^ (v1.6.6.0 and v1.6.0.9 for the proteome/phosphoproteome and in vitro kinase assays respectively), searching against the Human UniProt database (January, 2019), using default settings with the addition of ‘Phospho(STY)’ as a variable modification. The ‘Match between runs’ option was turned on for all analyses.

### Phosphoproteomics data analysis

Data analysis was performed using Perseus^49^ and R software environments. Phosphopeptide intensities were transformed (log2), and significantly regulated phosphopeptides identified by ANOVA (adj. *p* < 0.05) and Dunnett’s post hoc test (*p* < 0.05 Ctr vs DOX, untreated vs inhibitors). Enrichment of gene ontology annotation (GOBP, GOMF, GOCC, KEGG, Pfam) and enrichment of kinase-substrate motifs (PhosphoSitePlus)^50^ was performed using a Fisher exact test with Benjamini-Hochberg used for truncation (FDR < 0.02). Motif analysis was performed of phosphopeptides regulated in both treatments using IceLogo^51^. KinasePA^52^ was used to infer kinase activity in treatments vs controls^53^.

### siRNA transfection

Cells (5 × 10^5^) were transfected with 5 nM control, DYRK1A, RB1, *RBL2*, CDC23 siRNA and Lipofectamine RNA iMAX, according to manufacturer’s instructions (Life Technologies).

### Clonogenic assay

Cells (U251, 3 × 10^3^; HW1 and MMK1, 6 × 10^3^) were transfected with DYRK1A and control siRNAs, or treated with ALG for 14 days, fixed and stained with 1% Toluidine Blue (Sigma Aldrich). Colonies were counted using the ImageJ software (colony area plugging) and normalized to untreated controls.

### Antibodies

Antibodies used were from Cell Signaling (Danvers, MA, USA) against DYRK1A, P107, P16, p-RB, RB, p-CycD1, CycD1, CycB1, p-CDK1, Ki67, GAPDH, anti-rabbit HRP-linked, anti-mouse HRP-linked; from Abcam (Cambridge, UK) against CDK1, p-CDK1, CDC23, LIN52; and from Santa Cruz Biotechnology (Dallas, TX, USA) against LIN37, DYRK1A, LIN9, P130.

### Immunoblotting

Protein concentrations were determined with Pierce BCA assay kit (ThermoFisher Scientific #23225). Here, 20–40 μg of total protein was resolved on 4–12% SDS-PAGE gels and transferred onto PVDF membranes using iBlot 2 (all Life Technologies). Blocking with 5% skim milk (RT, 1 h) was followed by overnight incubation at 4 °C with primary antibodies in 5% BSA in TBS-T. Membranes were incubated with secondary antibodies in 1% skim milk in TBS-T (RT, 1 h). Detection was performed with Immobilon Western HRP Substrate Luminol-Peroxidase reagent (Merck Millipore) and the ChemiDoc MP Imaging System (Bio-Rad, Hercules, CA, USA). Densitometry quantification was done with ImageLab software (Bio-Rad).

### Immunoprecipitation

Cell pellets were homogenized in IP buffer and incubated with Protein A/G Sepharose (Life Technologies; 1 h, 4 °C). The precleared supernatants containing 1 mg of protein were incubated with P130 antibody overnight at 4 °C, then with Protein A/G Sepharose (3 h, 4 °C). Beads were washed with IP buffer and resuspended in NuPAGE LDS Sample Buffer and NuPAGE Sample Reducing Agent (Life Technologies). Bound proteins eluted from the immune complexes were denatured (5 min, 95 °C) and analysed by immunoblotting.

### Cycloheximide chase assay

Cells (5.0 × 10^5^) were transfected with DYRK1A and control siRNAs, or treated with ALG for 72 h, then treated with cycloheximide (30 μg/mL), lysed with RIPA buffer and analysed by immunoblotting.

### Flow cytometry

HW1 and MMK1 cells (1.0 × 106) were transfected with DYRK1A and Ctr siRNA (72 h), fixed and stained with propidium iodide (50 μg/mL) in the presence of RNase A (100 μg/mL, both Sigma Aldrich) for 1 h at 37 °C. Samples were analysed using LSRFortessa X-20 cytometer running FACSDiVa v6 software (BD Biosciences, Franklin Lakes, NJ, USA) and FlowJo v10.3 software.

### Immunofluorescence

Cells were fixed with ice-cold 4% PFA (20 min, RT), blocked in 5% normal goat serum/PBS (20 min) and incubated with anti-α-tubulin (1:10) and Ki67 (1:400) antibodies. Secondary antibodies were Alexa488-conjugated anti-mouse IgG and Alexa594-conjugated anti-rabbit IgG both (Life Technologies). Cell nuclei were counterstained using Prolong Gold mounting media with DAPI (Life Technologies). Images were acquired with a Zeiss Axio Scope.A1 microscope using ZEN 2 – blue edition software (Zeiss). Images were processed using Fiji.

### Nuclear staining

Cells were stained with NUCLEAR-ID Red DNA stain (30 min, 37 °C, 5% CO_2_), washed and mounted with Dako Fluorescence Mounting Medium (ENZO, Farmingdale, NY, USA). Images were acquired on a Zeiss upright fluorescence Axio Scope.A1 microscope and analysed using ImageJ. In each replicate, at least nine randomly chosen images were taken and number of nuclei quantified after setting the threshold automatically, using Li filter and ‘Analyse particle’ function in Batch mode.

### RT-PCR

Total RNA from cells was extracted using RNeasy mini kit (Qiagen, Hilden, Germany), retrotranscribed and amplified using Applied Biosystems High-Capacity cDNA Reverse Transcription kit (Life Technologies) as per manufacturer’s instructions. RT-PCR was performed using KAPA SYBR FAST Universal 2X qPCR Master Mix and Qiagen QuantiTect Primer Assays using standard procedures in a LightCycler 480. Threshold cycles (Ct) were calculated using the LightCycler^®^ 480 software (all Roche, Basel, Switzerland). Relative quantification using the comparative Ct method was used to analyse the data output. Values were expressed as fold change over corresponding values for the control by the 2-ΔΔCt method. Primers from QIAGEN: *GAPDH*, *CDKN1A*, *ORC1*, *MCM5*, *NEK2*, *BUB1*, *N2RF1*. Customized primers (Integrated DNA Technologies, Coralville, IO, IA): *p27* (fwd: 5′-CTG ATG CTG TTG CTC GGT TA-3′; rv: 5′-TGC AGA CTC TGG GAC ATC TG-3′), *DEC* (fwd: 5′-GGT TAG CGG AGC AAT GCG CA-3′; rv: 5′-AAC CGG CAT TTG GGG AAC CGT C-3′).

### Statistical analysis

All assays were performed in at least three independent experiments, values are expressed as mean ± SEM. Statistical comparisons were performed with GraphPad Prism v7 software, the null hypothesis was rejected at the 0.05 level. No statistical methods were used to pre-determine sample size but our sample sizes are equivalent to those reported in previous similar publications.

## Supplementary information

Supplementary Figure Legends.

Figure S1.

Figure S2.

Figure S3.

Figure S4.

Regaents_Tool.

Table S1.

Table S2.

Table S3.

## Data Availability

RAW and MaxQuant processed proteomics and phosphoproteomics data have been deposited to the PRIDE ProteomeXchange repository (https://www.ebi.ac.uk/pride/login), with the accession PXD020441, username reviewer52279@ebi.ac.uk and password *pu5hV07C*. Processed data are provided in the Supplementary Tables.
