# Supplementary material for: Global phosphoproteomics reveals DYRK1A regulates CDK1 activity in glioblastoma cells
Source: Cell Death Discov. 2021 Apr 16;7:81. doi: 10.1038/s41420-021-00456-6 (PMC8052442; doi:10.1038/s41420-021-00456-6)
Supplement: Supplementary file 1 — Supplementary Figure Legends. [file 41420_2021_456_MOESM1_ESM.docx]

**Supplementary Figures**

**Figure S1. Proteomics correlations and heatmaps.**

**(A)** Dots plots showing the average Pearson correlation of the proteome between all samples for DYRK1A pharmacological inhibition (left) and DYRK1A genetic knockdown (right). **(B)** Heapmat of overall protein changes in U251 (median log2 fold change of protein LFQ intensities for treatments vs their respective controls) after DYRK1A pharmacological inhibition (left) and DYRK1A genetic knockdown (right)

**Figure S2. Phosphoproteomics correlations and heatmaps.**

**(A)** Dot plots showing the average Pearson correlation of the phosphoproteome between all samples for DYRK1A pharmacological inhibition (left) and DYRK1A genetic knockdown (right). **(B)** Heatmap of overall phosphopeptides intensities in U251 (median log2 fold changes of protein LFQ intensities for treatments vs their respective controls) after DYRK1A pharmacological inhibition (left) and DYRK1A genetic knockdown (right). **(C)** Analysis of the amino acid sequences surrounding phosphorylated residues, revealing a strong preference for Proline (P) in the +1 position

**Figure S3. DYRK1A in vitro kinase assay.**

Raw MS/MS data showing CDC23 phosphorylation by DYRK1A in *in vitro* kinase assays using recombinant proteins **(A)** and in U251 phosphoproteomics experiments **(B).**

**Figure S4. Microscopic images illustrating reversibility of MMK1 cell dormancy.** Removal of colchicine from the media (drug holidays) allows MMK1 cells to recover their normal morphology and start proliferating again.
