# Supplementary figures and images for: Global phosphoproteomics reveals DYRK1A regulates CDK1 activity in glioblastoma cells
Source: Cell Death Discov. 2021 Apr 16;7:81. doi: 10.1038/s41420-021-00456-6 (PMC8052442; doi:10.1038/s41420-021-00456-6)

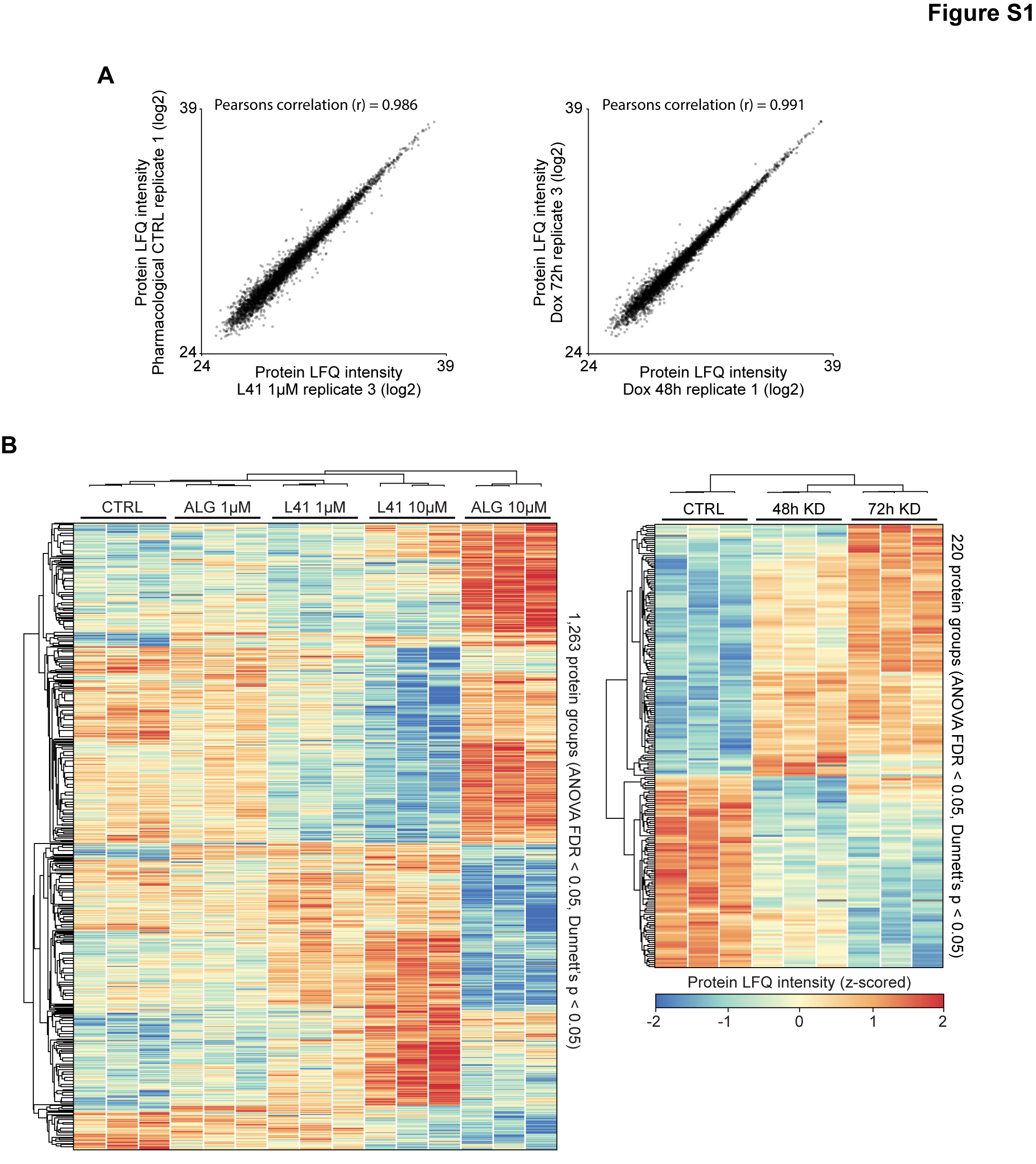

Supplement: Supplementary file 2 — Figure S1. [file 41420_2021_456_MOESM2_ESM.tif]

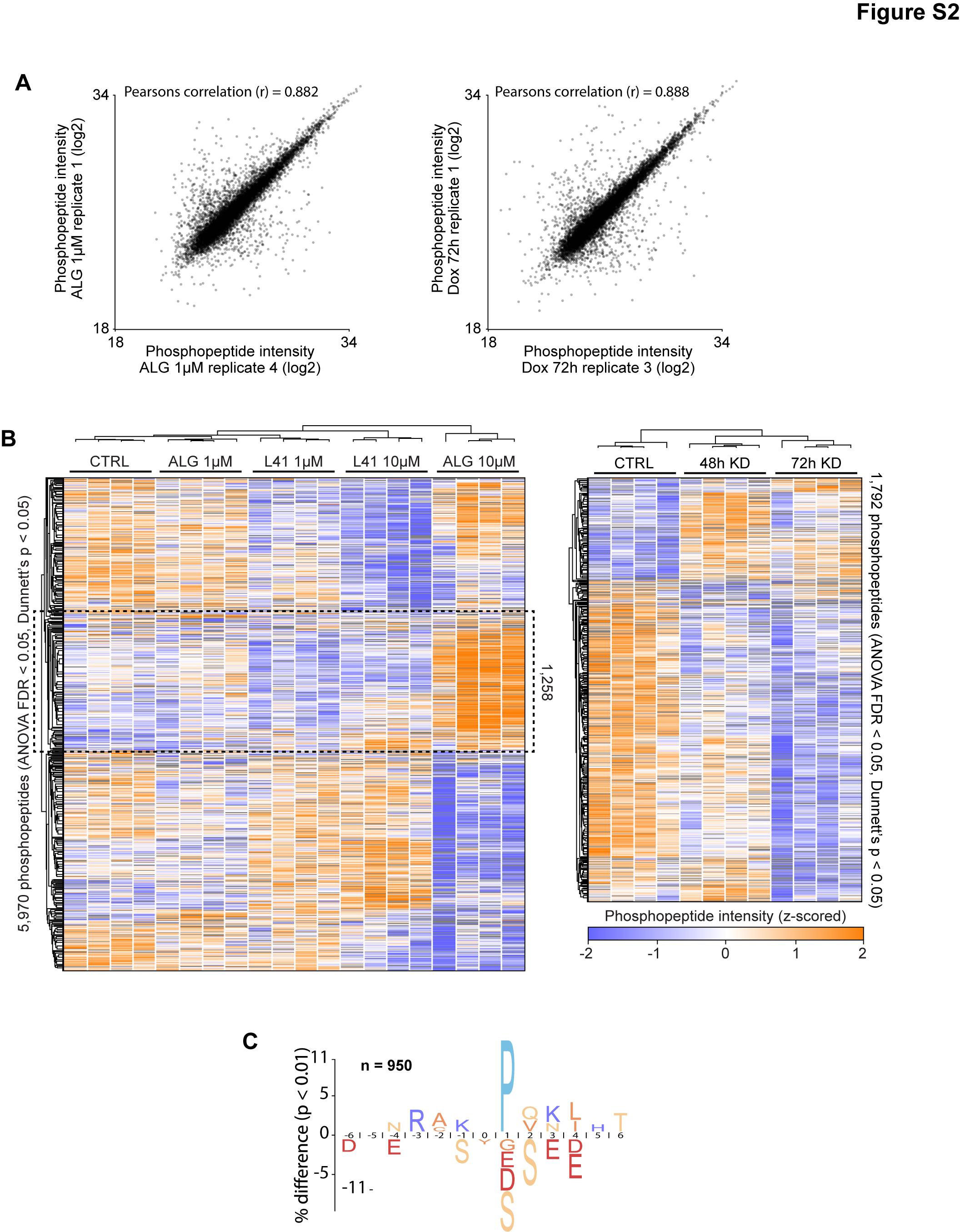

Supplement: Supplementary file 3 — Figure S2. [file 41420_2021_456_MOESM3_ESM.tif]

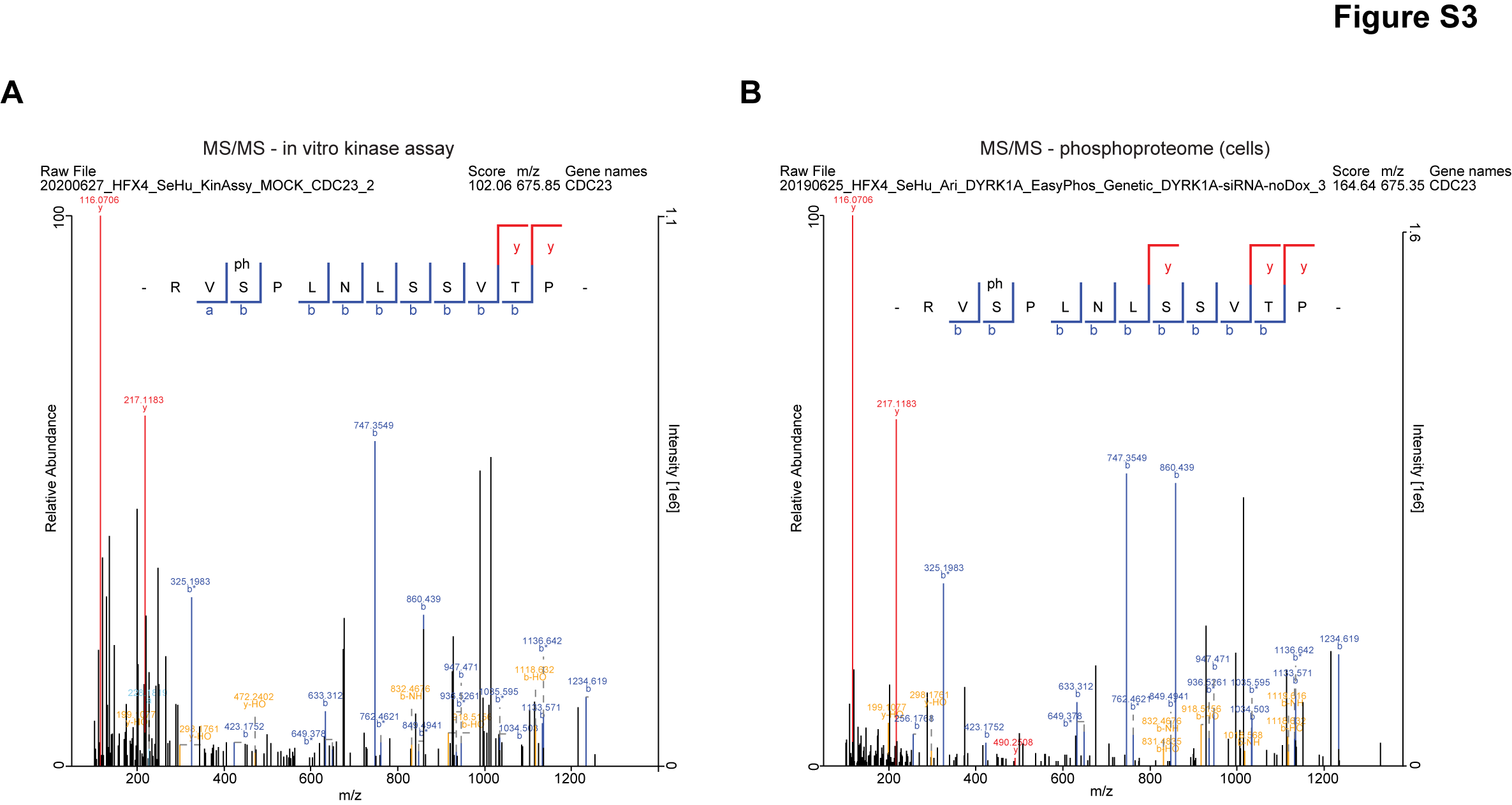

Supplement: Supplementary file 4 — Figure S3. [file 41420_2021_456_MOESM4_ESM.tif]

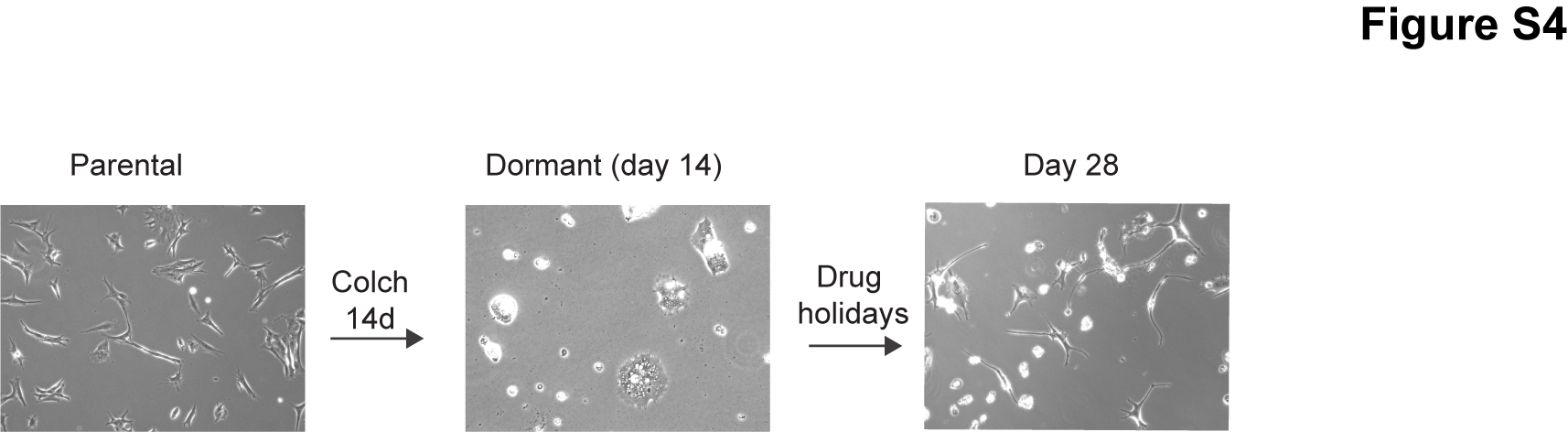

Supplement: Supplementary file 5 — Figure S4. [file 41420_2021_456_MOESM5_ESM.tif]
