# Supplementary material for: Global phosphoproteomics reveals DYRK1A regulates CDK1 activity in glioblastoma cells
Source: Cell Death Discov. 2021 Apr 16;7:81. doi: 10.1038/s41420-021-00456-6 (PMC8052442; doi:10.1038/s41420-021-00456-6)
Supplement: Supplementary file 6 — Regaents_Tool. [file 41420_2021_456_MOESM6_ESM.docx]

| **Reagent/Resource** | **Reference or Source** | **Identifier or Catalog Number** |
| --- | --- | --- |
| **Experimental Models** |  |  |
| A172 | ECACC | 88062428 |
| U251 | ECACC | 09063001 |
| HW1 | QMRI Berghofer Medical Research Institute | QIMR-B003 |
| MMK1 | QMRI Berghofer Medical Research Institute | QIMR-B004 |
| RKI1 | QMRI Berghofer Medical Research Institute | QIMR-B008 |
| RN1 | QMRI Berghofer Medical Research Institute | QIMR-B009 |
| **Recombinant DNA for sh DYRK1A-inducible U251** |  |  |
| shDYRK1A 1  Sense: 5’-tcccGTTCGGCTTGCACCGTCATTTCtcgaGAAATGACGGTGCAAGCCGAACttttttc-3’  Antisense: 5’- tcgagaaaaaaGTTCGGCTTGCACCGTCATTTCtcgaGAAATGACGGTGCAAGCCGAAC-3’ | Sigma Mission | shRNA TRCN0000273284 for pFH1t-II |
| shDYRK1A 1  Sense: 5’- tcccGCACAGATAGAAGTGCGACTTCtcgaGAAGTCGCACTTCTATCTGTGCttttttc-3’  Antisense: 5’- tcgagaaaaaaGCACAGATAGAAGTGCGACTTCtcgaGAAGTCGCACTTCTATCTGTGC-3’ | Sigma Mission | shRNA TRCN0000273347 for pFH1t-II |
| **Antibodies** |  |  |
| Rabbit polyclonal anti-DYRK1A (1/1000) | Cell Signaling Technology | Cat# 2771 |
| Mouse monoclonal anti-DYRK1A (1/1000) | Abcam | Cat# sc-100376 |
| Mouse monoclonal anti-LIN37 (1/1000) | Santa Cruz Biotechnology | Cat# sc-515686 |
| Mouse monoclonal anti-LIN9 (1/1000) | Santa Cruz Biotechnology | Cat# sc-130571 |
| Mouse monoclonal anti-P107 (1/1000) | Cell Signaling Technology | Cat# sc-250 |
| Mouse monoclonal anti-P130 (1/1000) | Santa Cruz Biotechnology | Cat# sc-374521 |
| Rabbit monoclonal anti-P16 (1/1000) | Cell Signaling Technology | Cat# 92803 |
| Rabbit policlonal anti-RB (phospho Ser807/811) (1/1000) | Cell Signaling Technology | Cat# 9308 |
| Mouse monoclonal anti-RB (1/2000) | Cell Signaling Technology | Cat# 9309 |
| Rabbit monoclonal anti-Cyclin D1 (phospho Thr286) (1/500) | Cell Signaling Technology | Cat# 3300 |
| Rabbit monoclonal anti-Cyclin D1 (1/500) | Cell Signaling Technology | Cat# 2978 |
| Mouse monoclonal anti-Cyclin B (1/1000) | Cell Signaling Technology | Cat# 4135S |
| Rabbit monoclonal anti-CDK1 (phospho Tyr15) (1/1000) | Cell Signaling Technology | Cat# 4539S |
| Mouse monoclonal anti-GAPDH (1/10000) | Cell Signaling Technology | Cat# 97166 |
| Rabbit monoclonal anti-CDK1 (1/1000) | Abcam | Cat# ab133327 |
| Rabbit monoclonal anti-CDK1 (phospho Thr161) (1/1000) | Abcam | Cat# ab201008 |
| Rabbit polyclonal anti-CDC23 (1/1000) | Abcam | Cat# ab72206 |
| Rabbit polyclonal anti-LIN52 (1/1000) | Abcam | Cat# ab115972 |
| Mouse monoclonal anti-b-Tubulin (1/10000) | Sigma-Aldrich | Cat# T8328 |
| Mouse monoclonal anti-Ki67 (1/500) | Cell Signaling Technology | Cat# 9449 |
| Goat anti-rabbit IgG, HRP-linked (1/5000) | Cell Signaling Technology | Cat# 7074 |
| Horse anti-mouse IgG, HRP-linked (1/5000) | Cell Signaling Technology | Cat# 7076 |
| Goat anti-mouse IgG, Alexa Fluor 488 (1/500) | Life Technologies | Cat# A10680 |
| Goat antirbbit IgG, AlexaFluor594 (1/500) | Life Technologies | Cat# A11012 |
| **Oligonucleotides and other sequence-based reagents** |  |  |
| Silencer Select Negative Control #1 siRNA | Life Technologies | Cat# 4390843 |
| Silence Select Pre-Designed DYRK1A siRNA  human NM_130438  Sense: GCUGACUACUUGAAGUUCATT  Antisense: UGAACUUCAAGUAGUCAGCGA | Life Technologies | Cat# 4390824, siRNA ID: s4401 |
| Silence Select Pre-Designed *RB1* siRNA  Human NM_000321  Sense: GCGUGUAAAUUCUACUGCATT  Antisense: UGCAGUAGAAUUUACACGCGT | Life Technologies | Cat# 4390824, siRNA ID: s522 |
| Silence Select Pre-Designed *RBL2 (p130)* siRNA  Human NM_005611  Sense: CACUAACUGGUGUUAGGUATT  Antisense: UACCUAACACCAGUUAGUGGT | Life Technologies | Cat# 4392420, siRNA ID: s11855 |
| Silence Select Pre-Designed *CDC23* siRNA  human NM_004661  Sense: GAUUGAUAAAUAUCGUGUATT  Antisense: UACACGAUAUUUAUCAAUCTC | Life Technologies | Cat# 4392420, siRNA ID: s16571 |
| QuantiTect Primer GAPDH | Qiagen | QT00079247 |
| QuantiTect Primer CDKN1A | Qiagen | QT00062090 |
| QuantiTect Primer PML | Qiagen | QT00090447 |
| QuantiTect Primer YPEL3 | Qiagen | QT00078589 |
| QuantiTect Primer ORC1 | Qiagen | QT00005341 |
| QuantiTect Primer MCM5 | Qiagen | QT00084000 |
| QuantiTect Primer NEK2 | Qiagen | QT00025221 |
| QuantiTect Primer BUB1 | Qiagen | QT00082929 |
| QuantiTect Primer NR2F1 | Qiagen | QT00089355 |
| Primer DEC | Integrated DNA Technologies | Forward: 5′-GGT TAG CGG AGC AAT GCG CA-3′,  Reverse: 5′-AAC CGG CAT TTG GGG AAC CGT C-3′ |
| Primer P27 | Integrated DNA Technologies | Forward Primer: 5′-CTG ATG CTG TTG CTC GGT TA-3′, Reverse Primer: 5′-TGC AGA CTC TGG GAC ATC TG-3′ |
| **Chemicals, Enzymes and other reagents** |  |  |
| Colchicine | Tocris | Cat# 1364, CAS: 64-86-8 |
| Leucettine L41 | Sapphire Bioscience | Cat# G-MR-C0023, CAS: 1112978-84-3 |
| Doxycycline | Sigma-Aldrich | Cat# D9891, CAS: 24390-14-5 |
| DAPI | Sigma-Aldrich | Cat# 32670, CAS: 28718-90-3 |
| Propidium iodide | Sigma-Aldrich | Cat# P4170 |
| Lipofectamine RNAiMAX | Life Technologies | Cat# 13778150 |
| Protein G Sepharose® 4 Fast Flow | GE Healthcare | Cat# 17-0618-01 |
| NUCLEAR-ID® Red DNA stain | ENZO | Cat# ENZ-52406 |
| Antibiotic/anti-mycotic | Life Technologies | Cat# 15240112 |
| Foetal bovine serum | InterPath | Cat# SFBS-f |
| Bovine serum albumin | Sigma-Aldrich | Cat# 3059, CAS: 9048-6-8 |
| Normal goat serum | Sigma-Aldrich | Cat# S-26 |
| Matrigel Matrix | Corning | Cat# 354234 |
| Prolong Gold Antifade Mountant with DAPI | Life Technologies | Cat# P36935 |
| Dako Fluorescence Mounting Medium | Agilent | Cat# S3023 |
| Complete™ Protease Inhibitor Cocktail | Roche | Cat# 11697498001 |
| Na_3_VO_4_ | Sigma-Aldrich | Cat# 450243, CAS: 13721-39-6 |
| Toluidine Blue | Sigma-Aldrich | Cat#: T3260 |
| Phenylmethanesulfonyl-fluoride (PMSF) | Sigma-Aldrich | Cat# 78830, CAS: 329-98-6 |
| ATP, [γ-32P]- 3000Ci/mmol 10mCi/ml EasyTide, 100 µCi | PerkinElmer | Cat# BLU502A100UC |
| Cdc23 Recombinant Protein | Novus Biological | Cat# H00008697 |
| KnockOutTM DMEM/F-12 medium | Life Technologies | Cat# 12660012 |
| StemProTM Neural Supplement | Life Technologies | Cat# A10508-01 |
| FGF-basic (AA 10–155) Recombinant Human  Protein | Life Technologies | Cat# PHG0024 |
| EGF Recombinant Human Protein | Life Technologies | Cat# PHG0314 |
| DMEM, high glucose, GlutaMAX™ Supplement, HEPES | Life Technologies | Cat# 10564011 |
| Protein A/G Agarose | Life Technologies | Cat# 20421 |
| Ribonuclease A | Sigma-Aldrich | Cat# R6513 |
| **Software** |  |  |
| Prism v7.0 | GraphPad | <https://www.graphpad.com/scientific-software/prism/> |
| ImageLab v6.0.1 | Bio-Rad | <http://www.bio-rad.com/en-au/product/image-lab-software?ID=KRE6P5E8Z> |
| FlowJo v10.3 | FlowJo | <https://www.flowjo.com/solutions/flowjo/downloads> |
| Fiji (ImageJ) | ImageJ | <https://imagej.net/Fiji/Downloads> |
| LightCycler 480 | Roche | <https://lifescience.roche.com/en_au/products/lightcycler14301-480-software-version-15.html> |
| MaxQuant | MaxQuant | <https://www.maxquant.org/download_asset/maxquant/latest> |
| Perseus | MaxQuant | <https://www.maxquant.org/download_asset/perseus/latest> |
| R | R project | <https://www.r-project.org/> |
| **Other** |  |  |
| example: Illumina NextSeq 500 | Illumina |  |
| Pierce BCA protein assay kit | ThermoFisher Scientific | Cat# 23225 |
| Immobilon Western HRP Substrate Luminol Peroxidase | MerckMillipore | Cat# WBKLS0500 |
| NuPAGE™ MOPS SDS Running Buffer (20X) | ThermoFisher Scientific | Cat# NP0001 |
| NuPAGE™ Sample Reducing Agent (10X) | ThermoFisher Scientific | Cat# NP0009 |
| NuPAGE™ LDS Sample Buffer (4X) | ThermoFisher Scientific | Cat# NP0007 |
| RNeasy mini kit | Qiagen | Cat# 74104 |
| Applied Biosystems High-Capacity cDNA Reverse Transcription kit | Life Technologies | Cat# 4368814 |
| KAPA SYBR FAST Universal 2X qPCR Master Mix | Kapa Biosystems | Cat# KK4602 |
| Bond Polymer Refine Detection Kit | Leica Microsystems | Cat# DS9800 |
| Optiphase SuperMix Scintillation Cocktail | PerkinElmer | Cat# 1200-439 |
